# Gene expression differences in *Bemisia tabaci* following acquisition of an Old World begomovirus

**DOI:** 10.1038/s41597-025-06417-3

**Published:** 2025-12-13

**Authors:** Zachary Lahey, Alvin M. Simmons, Sharon A. Andreason

**Affiliations:** United States Department of Agriculture, Agricultural Research Service, U.S. Vegetable Laboratory, Charleston, South Carolina 29414 USA

**Keywords:** Entomology, Non-model organisms, Transcriptomics

## Abstract

Begomoviruses (Geminiviridae) are economically important, high consequence plant viruses transmitted by members of the *Bemisia tabaci* (Gennadius) (Hemiptera: Aleyrodidae) cryptic species complex. One of the most economically significant begomoviruses is tomato yellow leaf curl virus (TYLCV), which is efficiently transmitted by *B*. *tabaci* Middle East–Asia Minor 1 (MEAM1), one of the most destructive agricultural pests globally. The purpose of this study is to determine the gene expression profiles of non-viruliferous and viruliferous MEAM1 after TYLCV acquisition access feeding. Adult whiteflies were fed on healthy or TYLCV infected tomato plants for 12, 36, or 60 hours, followed by a 12-hour gut clearing period on collard, a TYLCV non-host. Differential expression analyses (read mapping and transcript quantification) from RNA-seq data derived from ribodepleted total RNA were performed to determine gene expression patterns associated with TYLCV acquisition. In total, 37 differentially expressed genes were identified, including two horizontally acquired from plants. Elucidating the transcriptional response of MEAM1 to virus acquisition can inform the development of novel genomics-assisted whitefly transmitted virus management strategies.

## Background & Summary

Since its description as a pest of tobacco in Greece^1^, the whitefly *Bemisia tabaci* (Hemiptera: Aleyrodidae) has been a persistent scourge to field and greenhouse-grown agricultural crops, fibre commodities, and ornamental plants around the world^2^. In the United States, the 1980’s invasion of *B*. *tabaci* B biotype^3^, now often referred to as the Middle East–Asia Minor 1 (MEAM1)^4^ (also mitotype B or *Bemisia argentifolii* Bellows & Perring^5,6^), resulted in hundreds of millions of dollars in economic losses. Plant damage associated with rapid increases in the whitefly population, extreme polyphagy^7^, physiological disorders due to direct feeding^8,9^, and, most seriously, virus transmission^10^, were attributed to the new invader. Approximately 40 years later, *B*. *tabaci* continues to pose substantial problems for agriculturalists and subsistence farmers throughout the world, mainly due to the transmission of viruses, including begomoviruses (family *Geminiviridae*) which severely limit yield in numerous vegetable^11^, root^12^, and fibre crops^13^.

Begomoviruses are dicotyledonous plant viruses with single-stranded, mono- or bipartite circular genomes approximately 2.7 kilobases in length^14^. Viruses in this genus are spread exclusively by members of the *B*. *tabaci* cryptic species complex in a persistent, circulative manner^15^. Differential transmission of certain begomoviruses has been reported for different species within the complex^16–18^. The route viral particles take within the whitefly vector is complex^19^. The whitefly uses its stylets to ingest virions from phloem sap of an infected host, which then pass through the food canal, cibarium, and esophagus before entering the midgut filter chamber. The filter chamber is the main site where virions concentrate and are translocated into the haemolymph^20–22^. Once in the haemolymph, viral particles are protected en route to the primary salivary glands by complexing with a GroEL chaperone protein manufactured by the whitefly endosymbiotic bacterium *Hamiltonella* in MEAM1^23,24^ and *Arsenophonus* in Asia II-1 (also referred to as biotype/mitotypes K, P, ZHJ2, PCG-1, SY, PK1^4,25^). Virions are then internalized into the salivary glands and finally egested back into the plant phloem, where the cycle starts anew.

One of the most serious viral pathogens transmitted by *B*. *tabaci* is tomato yellow leaf curl virus (TYLCV)^26^, a monopartite begomovirus that causes tomato yellow leaf curl disease (TYLCD)^27^. The onset and severity of TYLCD symptoms are highly variable and dependent on the tomato cultivar, timing of inoculation relative to plant senescence, and environmental conditions. Typical TYLCD symptoms include upward curling and yellowing of leaflet margins, reduced size of leaflets, stunting, aborted blooms, reduced fruit size, and irregular fruit ripening^8,28^. Complete yield losses due to TYLCD are not uncommon^27^. Due to this notoriety, TYLCV is one of the most studied begomoviruses transmitted by *B*. *tabaci*, and developing effective management strategies to reduce TYLCV-induced economic losses is an active area of research^29–31^.

A promising research avenue to reduce the deleterious effects of TYLCV on tomato production is the adoption of genomics-based technologies. This has manifested as the identification of genetic loci that confer resistance to TYLCV and their integration into stable tomato breeding lines^32–34^, as well as the development of RNA interference (RNAi) technologies targeting genes in the vector that affect different life history parameters (e.g., longevity, fecundity, etc.) and virus transmission^35–38^. Target genes for RNAi may be identified through transcriptomic studies that evaluate the response of the whitefly to begomovirus acquisition. This is accomplished by measuring the number of differentially expressed genes (DEGs) between viruliferous and non-viruliferous whiteflies after they are allowed to feed on a host with or without the pathogen of interest. Coupled with a well-annotated genome assembly^39–41^, the DEGs can be identified, and their functions determined, to select the best candidates for downstream control applications (e.g., RNAi, CRISPR)^42,43^.

Several studies have examined differential gene expression in *B*. *tabaci* following begomovirus acquisition^44–57^, but relatively few have investigated the transcriptional response of *B*. *tabaci* MEAM1 to TYLCV acquisition. Those that have reported substantially different numbers of DEGs, which is most likely a result of key methodological differences including the use of propagative material (i.e., infected plant cuttings) instead of potted plants^49^, the sequencing of only one tissue type (e.g., gut tissue)^48^, and the implementation of a gut-clearing step following the TYLCV acquisition access period (AAP)^54^. In this study, we examined the transcriptional response of *B*. *tabaci* to TYLCV after three AAPs (12, 36, and 60 h) on potted tomato followed by a gut-clearing step on a TYLCV non-host, collard (*Brassica oleracea*). The gut-clearing step was implemented to minimize any potential indirect effects that feeding on infected plant tissue has on the vector^44,54^, thereby increasing the likelihood that changes in gene expression profiles between viruliferous and non-viruliferous whiteflies directly reflect virus-vector interactions. We also took a novel approach to preparing the sequenced RNA-seq libraries as compared to previous studies of the vector transcriptional response to begomovirus acquisition. To date, rRNA depleted RNA-seq libraries have not been reported in similar studies of whitefly-virus interactions. Previous studies have demonstrated that gene quantification variation is dependent on RNA sample preparation, with rRNA depletion able to capture more unique and rare transcripts than poly(A) selection^58–60^. Such transcripts include those encoding replication-dependent histone proteins^61^ as well as long-noncoding RNAs^62^. This dataset will be useful to researchers interested in studying novel *B*. *tabaci* genetic targets for developing enhanced genomics-informed whitefly and whitefly-transmitted virus management.

## Methods

### Whitefly colony

A colony of *B*. *tabaci* MEAM1 was established in 2022 from adult whiteflies collected on field grown zucchini (*Cucurbita pepo* L.) in the research fields of the Coastal Research and Education Center in Charleston, South Carolina, USA. Whiteflies were then reared on collard (*Brassica oleracea* var. acephala) in a greenhouse (26 ± 4 °C) at the USDA-ARS, U. S. Vegetable Laboratory, with fresh collard plants added to the colony as needed.

### Plant inoculations

To produce viruliferous whiteflies for use in plant inoculations, approximately 20 mating pairs of the MEAM1 colony maintained on collard were placed in clip cages and transferred to visibly symptomatic tomato plants (*Solanum lycopersicum* L., cultivar ‘Moneymaker’) infected with TYLCV for a 72-hour acquisition access period (AAP). Healthy tomato plants with at least three fully expanded true leaves were then subjected to feeding by approximately 20 mating pairs of non-viruliferous (healthy) or TYLCV-viruliferous (virus) whiteflies in clip cages for a 72-hour inoculation access period (IAP) to generate mock-inoculated (whitefly-exposed) and TYLCV-infected plants for use in experiments 3–4 weeks later. Plant infection status was assessed with end-point PCR before the initiation of feeding assays using primer pair KL14-324 (5′-CTTCGACAGCCCATACAGCA-3′) and KL14-325 (5′-GAGGGCCCACCAATAACTGT-3′) designed by Dr. Daniel Hasegawa in the laboratory of Dr. Kai-Shu Ling (USDA, ARS, U.S. Vegetable Laboratory, Charleston, SC, USA).

### Feeding assays

To acquire tomato-acclimated, age-specific (staged) whiteflies for use in experiments, older collard leaves from the whitefly colony bearing 4th instar whitefly nymphs, with adult whiteflies removed, were excised and added to cages containing virus-free tomato plants for a five-day emergence period. On day five, the emerged adult whiteflies were aspirated into standard insect collection vials and released onto the mock-inoculated or TYLCV-infected tomato plants. Approximately 1,500 adult whiteflies (≤ five days post-emergence) were added to each experimental plant, which were held in separate BugDorm cages (60 × 60 × 60 cm; MegaView Science Co., Ltd., Taichung, Taiwan) in an insectary maintained at 26 ± 4 °C, 45 ± 15% RH, and a natural light/dark cycle (14:10 L:D). Whiteflies were allowed to feed for three acquisition access periods (AAPs): 12, 36, and 60 hours. At the end of each AAP, approximately 500 adult whiteflies from each experimental cage were transferred to collard, a non-host of TYLCV, for a 12-hr gut clearing period. The gut clearing step was added to ensure that differential expression was due to virus ingestion and acquisition rather than a response to feeding on TYLCV-infected plant tissue. Following gut clearing, all whiteflies were collected and frozen at −80 °C until RNA extraction. The experiment was replicated three times, and each replication consisted of one plant per treatment.

### RNA extraction, Illumina library preparation, and RNA sequencing

Total RNA was extracted from whiteflies using TRIzol® with the PureLink® RNA Mini Kit following the manufacturer’s recommended protocol, except that initial sample lysis was conducted in 100 µL TRIzol® using an ice-cold pestle and the optional on-column DNase treatment protocol was performed. Illumina library preparation and RNA sequencing were performed at the Genomics Core Facility of Michigan State University (East Lansing, MI, USA). Whitefly and bacterial (symbiont) ribosomal RNA (rRNA) was depleted from samples using the QIAseq FastSelect rRNA Fly and 5S/16S/23S kits, respectively (Qiagen, Hilden, Germany). Stranded RNA-seq libraries were prepared using the Illumina Stranded Total RNA Library Preparation Ligation Kit (Illumina, Inc., San Diego, CA, USA) with Integrated DNA Technologies Unique Dual Index adapters following the manufacturer’s recommendations, except that half volume reactions were used. Library quality control and quantification was conducted using a combination of Qubit dsDNA HS (Thermo Fisher Scientific, Inc., Waltham, MA, USA) and Agilent 4200 TapeStation HS DNA1000 assays (Agilent Technologies, Santa Clara, CA, USA). The libraries were pooled in equimolar proportions and quantified using an Invitrogen Collibri Quantification qPCR kit (Thermo Fisher Scientific, Inc., Waltham, MA, USA). Sequencing was performed on an Illumina NovaSeq6000 sequencing system. The pool was loaded onto one lane of an Illumina S4 flow cell, and sequencing was performed in a 2×150bp paired-end format using a NovaSeq v1.5, 300 cycle reagent kit. Base calling was done by Illumina Real Time Analysis (RTA) v3.4.4, and the output of RTA was demultiplexed and converted to FASTQ format with Illumina Bcl2fastq (v2.20.0).

### RNA-seq read processing, mapping, and differential expression analyses

**Fig. 1:**
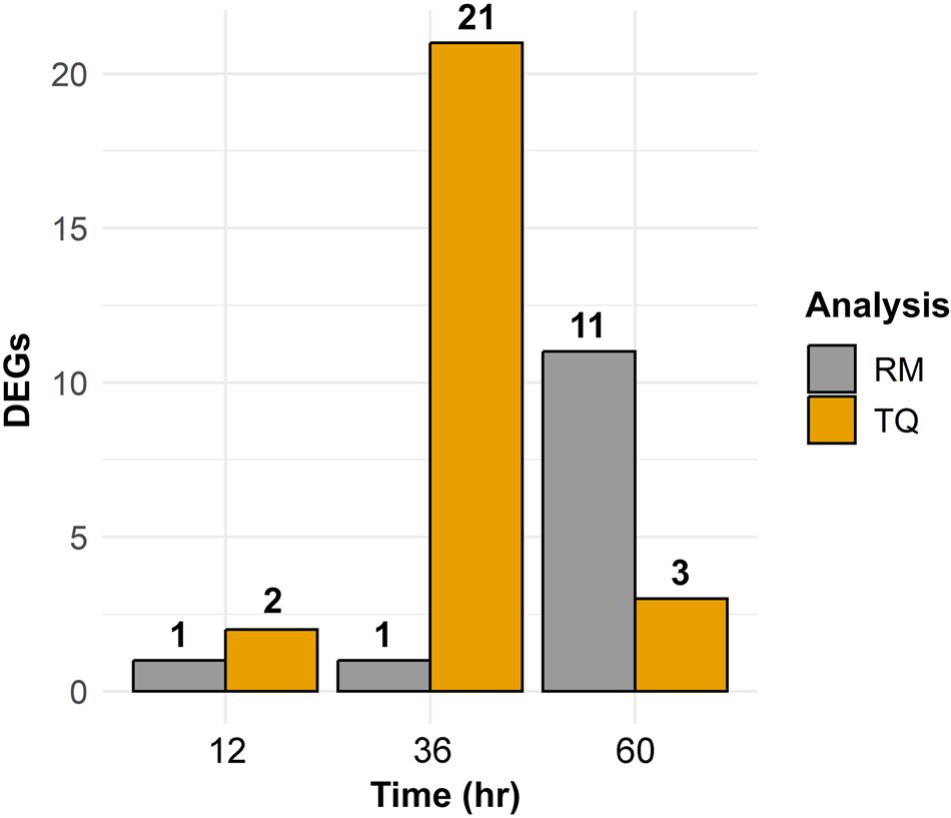
Bar chart of the number of differentially expressed genes (DEGs) at each acquisition access period (AAP) identified by read mapping (RM) and transcript quantification (TQ).

Raw reads from the 18 RNA-seq libraries (six treatments with three replicates) were processed with fastp^63^ (v.0.12.4) to remove sequencing adapters, trim low-quality bases, and remove reads less than 75 base pairs (bp) in length. The cleaned reads were then aligned to the MEAM1 mitochondrial genome (NCBI Reference Sequence: NC_006279.1), three bacterial endosymbiont genomes (*Candidatus* Portiera aleyrodidarum, *Candidatus* Hamiltontella defensa, and *Rickettsia* sp. available at http://www.whiteflygenomics.org/ftp/MEAM1/endosymbionts/), and Release 138.1 of the SILVA rRNA database (SSU and LSU NR 99 Ref sequences; accessed on 26 June 2023) using HISAT2^64^ (v.2.2.1). The aligned reads were filtered out, and the remaining reads were aligned to the MEAM1 nuclear genome assembly^39^ with STAR^65^ (v.2.7.10b) using the splice site information included in the MEAM1 general feature format file at http://www.whiteflygenomics.org/ftp/MEAM1/v1.2/MEAM1_v1.2.gff3.gz. The resulting SAM files were converted to BAM format with samtools^66^ (v.1.17). Differential expression (DE) analyses on read- and gene-level count matrices were performed with DESeq. 2^67^ (v.1.40.2) in R^68^. The count matrix of sequencing reads unambiguously mapped to a gene (hereafter referred to as read mapping; RM) was generated using the BAM files mentioned previously. The gene-level count matrix (hereafter referred to as transcript quantification; TQ) was created with Salmon^69^ (v.1.6.0) and imported to R using the *tximport* package^70^ (v.1.28.0). In both RM and TQ analyses, genes with an adjusted *p*-value < 0.1 and minimum fold change of 1.2 were considered as differentially expressed. The illustrations in Fig. 1 were created using the package ggplot2^71^ (v.4.4.1) in R.

On average, 32.5 million cleaned RNA-seq reads were generated per library (N = 18), and 96.1% of the cleaned reads in each library aligned to the MEAM1 nuclear genome assembly (Table 1). Approximately four million more RNA-seq reads were generated per library in viruliferous (36.6 million) vs non-viruliferous whiteflies (32.6 million).

**Table 1:** Summary of ribodepleted RNA-Seq datasets generated from *Bemisia tabaci* MEAM1 whiteflies fed for 12, 36, or 60 h on healthy (virus free) and tomato yellow leaf curl virus infected plants followed by 12 h of gut clearing on collard.

Sample	Replicate	Time point (h)	No. raw reads	No. cleaned reads	Unique cleaned reads mapped to MEAM1 genome	
					No. mapped	% mapped
Healthy	1	24	35,928,426	35,331,175	33,143,018	96.1
Healthy	2	24	39,861,824	39,173,860	36,526,020	95.5
Healthy	3	24	36,375,456	35,822,921	33,650,141	95.9
Virus	1	24	35,530,473	34,849,853	32,865,577	96.1
Virus	2	24	43,516,864	42,761,999	40,340,522	96.3
Virus	3	24	38,061,435	37,454,859	35,227,644	96.0
Healthy	1	48	31,185,688	30,657,396	28,931,176	96.3
Healthy	2	48	31,525,922	30,959,226	29,095,315	96.0
Healthy	3	48	26,624,019	26,138,485	24,401,684	95.8
Virus	1	48	35,975,471	35,238,885	33,170,598	96.2
Virus	2	48	27,320,951	26,743,614	25,389,193	96.7
Virus	3	48	36,044,527	35,324,760	33,348,155	96.3
Healthy	1	72	35,123,453	34,368,139	32,385,242	96.5
Healthy	2	72	26,687,888	26,126,516	24,503,720	96.2
Healthy	3	72	35,280,327	34,647,050	32,519,691	96.1
Virus	1	72	36,611,371	35,745,886	33,755,270	96.5
Virus	2	72	33,322,698	32,861,154	30,689,940	95.9
Virus	3	72	49,082,829	48,353,133	45,172,415	95.6

Thirty-seven DEGs were identified across all time points and types of analysis (Fig. 1; Table 2). Thirteen and 26 DEGs were identified by RM and TQ, respectively. Two genes (Bta05741, Bta05749) were identified as differentially expressed in both analyses, and no genes were differentially expressed at more than one time point. In RM analyses, 10 genes were upregulated, and 3 genes were downregulated. Two of the genes upregulated at 72 hours (Bta13103, Bta13961) were horizontally acquired from plants^72^. In TQ analyses, 8 genes were upregulated, and 18 genes were downregulated.

**Table 2:** Differentially expressed genes in *Bemisia tabaci* MEAM1 fed for 12, 36, and 60 h on healthy (virus free) and tomato yellow leaf curl virus infected plants followed by 12 h of gut clearing on collard. GeneIDs followed by an asterisk were identified as differentially expressed in both analyses.

GeneID	Time point (h)	Annotation	Fold Change	Adjusted P-value
**Read Mapping**				
Bta09316	24	None	1.23	0.087
Bta05759*	48	Unknown protein	1.46	0.039
Bta05741*	72	Unknown protein	1.91	0.000
Bta15312	72	Reverse transcriptase	−4.05	0.000
Bta13457	72	Unknown protein	1.66	0.001
Bta06687	72	Unknown protein	2.88	0.002
Bta01272	72	Histone H2A	−1.54	0.006
Bta13864	72	Unknown protein	2.30	0.009
Bta13103	72	rRNA N-glycosidase	1.44	0.014
Bta13961	72	Thaumatin-like protein 1a	1.72	0.014
Bta02118	72	Glucosylceramidase	1.49	0.069
Bta05685	72	Unknown protein	2.63	0.069
Bta02612	72	Protein kinase C	−1.29	0.098
**Transcript Quantification**				
Bta14861	24	D-alanine–poly(phosphoribitol) ligase subunit 1	−1.51	0.008
Bta04330	24	Unknown protein	−1.25	0.094
Bta03745	48	G/T mismatch-specific thymine DNA glycosylase	−1.23	0.041
Bta14135	48	ADP-ribosylation factor family	−1.24	0.041
Bta05213	48	Histone H2B	1.24	0.048
Bta05759*	48	Unknown protein	1.37	0.048
Bta07538	48	Cyclin-A1	−1.21	0.048
Bta08644	48	PAX-interacting protein	−1.25	0.048
Bta01987	48	Cytochrome c oxidase subunit 1	−0.77	0.049
Bta00576	48	Ubiquitin carboxyl-terminal hydrolase	−1.20	0.060
Bta08449	48	Ribosomal protein L30	−0.80	0.060
Bta00412	48	Double-stranded RNA-specific editase 1	−1.16	0.078
Bta02155	48	Histone H2B	1.20	0.078
Bta12553	48	Kinetochore protein NDC80-like protein	−1.28	0.078
Bta13581	48	Mastermind	−1.25	0.078
Bta02342	48	Peptidyl-prolyl cis-trans isomerase	1.30	0.081
Bta00196	48	CTAGE family member 5	−1.20	0.085
Bta03026	48	30S ribosomal protein S8	−1.21	0.086
Bta03449	48	Solute carrier family 35 member E1-like protein	−1.29	0.086
Bta11294	48	E3 ubiquitin-protein ligase TRIM37	−1.27	0.086
Bta12260	48	40S ribosomal protein S24	1.26	0.086
Bta13866	48	NADH dehydrogenase flavoprotein 1	1.25	0.089
Bta02577	48	E3 ubiquitin-protein ligase RNF139	−1.24	0.098
Bta05741*	72	Unknown protein	1.91	0.000
Bta13457	72	Unknown protein	1.94	0.001
Bta13640	72	Chemosensory protein	1.86	0.002

## Data Records

The data associated with this project have been accessioned with the National Center for Biotechnology Information (NCBI) under BioProject number PRJNA1096732: Transcriptomics of viruliferous and non-viruliferous *Bemisia tabaci* MEAM1^73^. Illumina RNA-seq reads have been deposited in the NCBI Sequence Read Archive under accession numbers SRR28578498–SRR28578515, and the BioSamples used to generate the RNA-seq reads have been assigned BioSample accession numbers SAMN40761531– SAMN40761536.

## Technical Validation

Total RNA quantification was performed using a DeNovix DS-11 FX spectrophotometer/fluorometer (DeNovix Inc., Wilmington, DE, USA) using 1 µL of total RNA. RNA integrity was assessed on a 2100 Bioanalyzer instrument (Agilent Technologies, Santa Clara, CA, USA) at the Molecular Analytics Core, part of the Department of Regenerative Medicine and Cell Biology at the Medical University of South Carolina (Charleston, SC, USA). Cohorts of six (non-viruliferous) and 10 (viruliferous) whiteflies from each replicate and time point were collected and individually extracted to screen for the absence or presence of TYLCV. All whiteflies in the non-viruliferous treatments tested negative for TYLCV at each time point. In the viruliferous treatments, 90%, 93%, and 97% of the whiteflies at 24, 48, and 72 hours, respectively, tested positive for TYLCV.

Select up- and downregulated genes from the RM and TQ DEG analyses were validated using a QIAcuity One digital PCR (dPCR) system (QIAGEN, Hilden, Germany) (Supplementary Table 1). Full-length coding sequences of each gene in the *B*. *tabaci* MEAM1 genome (version 1.2; Chen *et al*. 2016), downloaded at http://www.whiteflygenomics.org/ftp/MEAM1/v1.2/MEAM1_CDS_v1.2.fa.gz, were used to design primers with Primer3web^74^ (version 4.1.0) at default parameters (https://primer3.ut.ee/). Primers for each DEG are listed in Supplementary Table 2. dPCR Reactions consisted of 5 µL EvaGreen master mix (QIAGEN, Hilden, Germany), 0.6 µL 10 µM forward and reverse primers, 1.5 µL cDNA at 500 ng/µL, and 7.3 µL nanopure water. Reactions were performed in 24 or 96 well nanoplates with 8.5k partitions per well. Thermocycling consisted of an initial 2 min denaturation at 95 °C, 40 cycles at 95 °C for 30 s and 60 °C for 60 s, and a final extension for 10 min at 35 °C. Imaging was performed at an exposure duration of 200 ms and gain of 6, and the threshold for positive partitions was set at 50 RFU. The expression of each gene was normalized to the whitefly gene α-tubulin, a housekeeping gene.

## Supplementary information

Supplementary Material

## Data Availability

Datasets are available through the NCBI repository BioProject number PRJNA1096732 at https://www.ncbi.nlm.nih.gov/bioproject/PRJNA1096732.

## Code availability

A text file with example commands used to reproduce the analyses is included as a supplement.
