# Supplementary material for: Gene expression differences in Bemisia tabaci following acquisition of an Old World begomovirus
Source: Sci Data. 2025 Dec 13;13:103. doi: 10.1038/s41597-025-06417-3 (PMC12848113; doi:10.1038/s41597-025-06417-3)
Supplement: Supplementary file 1 — Supplementary Material [file 41597_2025_6417_MOESM1_ESM.pdf]

Supplementary Material

Supplementary Table 1. Differentially expressed genes validated with digital PCR.

GeneID	Annotation	Time Point (h)	Analysis	RNA-seq concordant	dPCR concordant (α-tubulin)
Bta14861	D-alanine--poly(phosphoribitol) ligase subunit 1	24	TQ	-0.66	-0.86
Bta02342	Peptidyl-prolyl cis-trans isomerase	48	TQ	1.30	1.60
Bta00196	CTAGE family member 5	48	TQ	-1.20	-0.94
Bta00412	Double-stranded RNA-specific editase 1	48	TQ	-1.16	-0.95
Bta00576	Ubiquitin carboxyl-terminal hydrolase	48	TQ	-1.20	-0.95
Bta03449	Solute carrier family 35 member E1-like protein	48	TQ	-1.29	-2.19
Bta03745	G/T mismatch-specific thymine DNA glycosylase	48	TQ	-0.98	-1.23
Bta13866	NADH dehydrogenase flavoprotein 1	48	TQ	1.25	1.60
Bta05685	Unknown protein	72	RM	2.63	1.80
Bta13961	Thaumatin-like protein 1a	72	RM	1.72	2.75
Bta13457	Unknown protein	72	RM/TQ	1.66	2.43
Bta13864	Unknown protein	72	RM	2.30	1.99

Supplementary Table 2. Digital PCR validation primers used in this study.

GeneID	Annotation	Forward Sequence (5'-->3')	Reverse Sequence (5'-->3')
Bta14861	D-alanine--poly(phosphoribitol) ligase subunit 1	CCACCCTCAATCGCAACAAA	CCTCCACCGCCTTTATTTCG
Bta02342	Peptidyl-prolyl cis-trans isomerase	GGAACTGGTGGTCGCAGTAT	CCAGGGGGTCTGTTTTGTGG
Bta00196	CTAGE family member 5	GTTGAAGCTCCAAACGCAGA	GCTGACTCGACAACTGCTTC
Bta00412	Double-stranded RNA-specific editase 1	AATCAGGCTAACACAACGGC	GGGTGTTGTAGTTGCCAGTG
Bta00576	Ubiquitin carboxyl-terminal hydrolase	AAAAGTACATCAGCGCCCAC	TCATTTCCTCCGCCAGAAGT
Bta03449	Solute carrier family 35 member E1-like protein	GGTGTACTAAGCTGGCTCCA	CCGAAAATATTGGTGCCCGT
Bta03745	G/T mismatch-specific thymine DNA glycosylase	CCTATGTACCCTAGCCAGCC	TGCTGCACTGATTTCGGATG
Bta13866	NADH dehydrogenase flavoprotein 1	AAGCGTTTGTGAGGATGTCC	TGCACTGACCACATGACTCA
Bta05685	Unknown protein	GGAGAGCGAGATTGAACCGA	CGCAACCGACTTGAGAAAGG
Bta13961	Thaumatin-like protein 1a	ACAACCACAACACCAGGGAG	GGCAGAAGGTGACGACGTAG
Bta13457	Unknown protein	GCGAGAAGATCACCAACGAG	ATCTTGTAGCAGCGGTTGAC
Bta13864	Unknown protein	AAGGATTTCAACGACCGCAC	CCCGATGGAGGTGAGCTTAT

#Supplementary material to accompany Lahey Z, Simmons AM, Andreason SA. Gene expression differences in Bemisia tabaci following acquisition of an Old World begomovirus. Submitted to Scientific Data.

```
>cat list.txt
H124
H224
H324
H148
H248
H348
H172
H272
H372
V124
V224
V324
V148
V248
V348
V172
V272
V372
```

#1 clean reads with fastp (performs trimming and adapter removal in one step)

```
for f in `cat list.txt`; do fastp -i $f\_R1_001.fastq.gz -I $f\_R2_001.fastq.gz \
-o $f\.R1.fq.gz -O $f\.R2.fq.gz --length_required 75 --thread 8 \
--html $f\.html; done
```

#2 filter out reads that align to the MEAM1 mitochondrial genome, endosymbiont genomes, and ribosomal RNA

```
hisat2-build mito_endo_ribo.fasta 3_hisat2/mito_endo_ribo
```

```
for f in `cat list.txt`; do hisat2 -x 3_hisat2/mito_endo_ribo -1 2_trimmed_reads/$f\.R1.fq.gz \
-2 2_trimmed_reads/$f\.R2.fq.gz \
-p 48 -S 3_hisat2/$f\.sam --un-gz 3_hisat2/$f\.un.fastq.gz --al-gz 3_hisat2/$f\.al.fastq.gz \
--un-conc-gz 3_hisat2/$f\.unconc.fastq.gz --al-conc-gz 3_hisat2/$f\.alconc.fastq.gz; done

#3 align filtered reads to MEAM1 genome

STAR --runThreadN 48 \
--runMode genomeGenerate \
--genomeDir /project/usvl_vector/endosymbiont_project/raw_reads \
--genomeFastaFiles MEAM1_scaffold_v1.2.fa \
--genomeSAindexNbases 13 \
--sjdbGTFfile MEAM1_v1.2.gtf \
--sjdbOverhang 149

for f in `cat list.txt`; do STAR --genomeDir /project/usvl_vector/endosymbiont_project/raw_reads \
--readFilesIn /project/usvl_vector/endosymbiont_project/raw_reads/3_hisat2/$f\.unconc.fastq.1.gz \
/project/usvl_vector/endosymbiont_project/raw_reads/3_hisat2/$f\.unconc.fastq.2.gz \
--outFilterScoreMinOverLread 0.3 --outFilterMatchNminOverLread 0.3 \
--readFilesCommand zcat --runThreadN 48 --outFileNamePrefix 4_star/$f\.; done

#4 convert sam to bam
for f in `cat list.txt`; do samtools view -bS 4_star/$f\.Aligned.out.sam \
-o 4_star/$f\.bam; done

#5 perform differential expression analyses
#5a Read Mapping (consult Love et al. 2014)
dir <- getwd()

csvfile <- file.path(dir,"sample_table.csv")

sampleTable <- read.csv(csvfile,row.names=1)

filenames <- file.path(dir, paste0(sampleTable$name, ".bam"))
```

```
library("Rsamtools")
bamfiles <- BamFileList(filenames, yieldSize=5000000)

library("GenomicFeatures")

gtffile <- file.path(dir,"MEAM1_v1.2.gtf")
(txdb <- makeTxDbFromGFF(gtffile, format="gtf", circ_seqs=character()))

(ebg <- exonsBy(txdb, by="gene"))

library("GenomicAlignments")

se <- summarizeOverlaps(features=ebg, reads=bamfiles,
mode="Union",
singleEnd=FALSE,
ignore.strand=FALSE,
fragments=TRUE )

colData(se) <- DataFrame(sampleTable)

se$trt <- factor(se$trt)
se$time <- factor(se$time)
se$name <- factor(se$name)

library("DESeq2")

dds <- DESeqDataSet(se, design = ~ trt)

nrow(dds)

dds <- dds[ rowSums(counts(dds)) > 1, ]
nrow(dds)
```

```
rld <- rlog(dds, blind=FALSE)
head(assay(rld), 3)

par( mfrow = c( 1, 2 ) )
dds <- estimateSizeFactors(dds)
plot(log2(counts(dds, normalized=TRUE)[,1:2] + 1),
    pch=16, cex=0.3)
plot(assay(rld)[,1:2],
    pch=16, cex=0.3)

dds <- DESeq(dds)

(res <- results(dds))

summary(res)

res.1 <- results(dds, alpha=.1)
table(res.1$padj < 0.1)

sum(res$pvalue < 0.1, na.rm=TRUE)
sum(!is.na(res$pvalue))

sum(res$padj < 0.1, na.rm=TRUE)

resSig <- subset(res, padj < 0.1)
head(resSig[ order(resSig$log2FoldChange), ])

head(resSig[ order(resSig$log2FoldChange, decreasing=TRUE), ])

#5a Read Mapping (consult Patro et al. 2017; Soneson et al. 2016)
dir <- getwd()

csvfile <- file.path(dir,"sample_table.csv")
```

```
sampleTable <- read.csv(csvfile,row.names=1)

files <- file.path(dir, sampleTable$name, paste0(sampleTable$name, ".quant.sf"))

names(files) <- paste0(sampleTable$name)

library("tximport")

txi.tx <- tximport(files, type = "salmon", txOut = TRUE)

library("DESeq2")

ddsTxi <- DESeqDataSetFromTximport(txi.tx,
                                   colData = sampleTable,
                                   design = ~ trt)

dds <- DESeq(ddsTxi)
res <- results(dds)
res

summary(res)

res.1 <- results(dds, alpha=.1)
table(res.1$padj < 0.1)

sum(res$pvalue < 0.1, na.rm=TRUE)
sum(!is.na(res$pvalue))

sum(res$padj < 0.1, na.rm=TRUE)
```
